# Evaluation of the Analytical Performance of Oncomine Lung cfDNA Assay for Detection of Plasma *EGFR* Mutations

**DOI:** 10.3390/genes14061219

**Published:** 2023-06-02

**Authors:** Yong Gon Cho, Joonhong Park, Ji Yoon Han, Tae Yun Kim

**Affiliations:** 1 Department of Laboratory Medicine, Jeonbuk National University Medical School and Hospital, Jeonju 54907, Republic of Korea; choyg@jbnu.ac.kr (Y.G.C.); miziro@jbnu.ac.kr (J.P.); 2 Research Institute of Clinical Medicine, Jeonbuk National University-Biomedical Research Institute, Jeonbuk National University Hospital, Jeonju 54907, Republic of Korea; 3 Department of Pediatrics, College of Medicine, The Catholic University of Korea, Seoul 06591, Republic of Korea; 4 Department of Thoracic and Cardiovascular Surgery, Jeonbuk National University Medical School and Hospital, Jeonju 54907, Republic of Korea

**Keywords:** analytical performance, Oncomine Lung cfDNA Assay, plasma *EGFR* mutations, next-generation sequencing, cobas^®^ *EGFR* Mutation Test v2

## Abstract

Background: The clinical utility of circulating tumor DNA (ctDNA) in the early detection of tumor mutations for targeted therapy and the monitoring of tumor recurrence has been reported. However, the analytical validation of ctDNA assays is required for clinical application. Methods: This study evaluated the analytical performance of the Oncomine Lung cfDNA Assay compared with the cobas^®^ *EGFR* Mutation Test v2. The analytical specificity and sensitivity were estimated using commercially pre-certified reference materials. The comparative evaluation of the two assays was carried out using reference materials and plasma derived from patients diagnosed with lung cancer. Results: Using 20 ng of input cell-free DNA (cfDNA), the analytical sensitivities for *EGFR* mutations with variant allele frequencies (VAFs) of 1% and 0.1% were 100% and 100%, respectively. With VAFs of 1.2% and 0.1% using 20 ng of input cfDNA, seven out of nine different mutations in six driver genes were identified in the Oncomine Lung cfDNA Assay. The two assays showed 100% concordance in 16 plasma samples clinically. Furthermore, various *PIK3CA* and/or *TP53* mutations were identified only in the Oncomine Lung cfDNA Assay. Conclusions: The Oncomine Lung cfDNA Assay can be used to identify plasma *EGFR* mutations in patients with lung cancer, although further large-scale studies are required to evaluate the analytical validity for other types of aberrations and genes using clinical samples.

## 1. Introduction

Circulating tumor DNA (ctDNA) reflects the mutation landscape of tumor tissues as a component of cell-free DNA (cfDNA) in patients with cancer. Assays that estimate genomic variants in ctDNA are required in the clinical oncology setting, despite uncertainties surrounding their pre-analytical and analytical clinical validity and utility [1]. Several studies have demonstrated the clinical utility of ctDNA in different cancer types for the early identification of tumors, the detection of mutations for targeted molecular therapy, and the monitoring of tumor recurrence [2,3]. For example, tyrosine kinase inhibitors (TKIs) show significant clinical benefit in patients with non-small cell lung cancer (NSCLC) harboring epidermal growth factor receptor (*EGFR*)-sensitizing mutations [4]. Various ultrasensitive methods for the detection of tumor genotypes, including mutant enrichment real time polymerase chain reaction (RT-PCR), droplet digital PCR (ddPCR), and next generation sequencing (NGS) play a significant role in the era of targeted therapy and personalized medicine for the management of patients with solid cancers [5,6]. The cobas^®^ *EGFR* Mutation Test v2 (Roche Molecular Systems, Pleasanton, CA, USA) is a rapid and reliable method with acceptable sensitivity for the analysis of plasma or formalin-fixed paraffin-embedded (FFPE) tissue samples. It is an RT-PCR technique for the determination of several hotspot *EGFR* mutations at the time of diagnosis and facilitates the evaluation of patients with NSCLC for EGFR-TKI therapy [7]. However, pre-analytical factors, such as the location of metastasis, tumor cell content, or tumor burden, should be evaluated to accurately interpret results reported by medical laboratories. In contrast to targeted analysis, the NGS-based approach has greatly improved the precision, specificity, and sensitivity of the detection of rare copy number variations (CNVs), single- or multiple-nucleotide variants, and/or fusion transcripts of several driver genes in simultaneous tests [8]. In addition, this analysis can achieve a 0.1% limit of detection when using specific strategies, such as computational post-processing [8], unique molecular identifiers for distinguishing true rare variants from sequencing artifacts [9], and a deeper depth of coverage [10].

Previous cross-platform comparisons between the cobas^®^ *EGFR* Mutation Test v2 and the Oncomine Lung cfDNA assay (Thermo Fisher Scientific, Waltham, MA, USA), which is designed to identify single-nucleotide variants (SNVs) and small indels from 11 driver genes, [11] or Oncomine Pan-Cancer Cell-Free Assay [12] have been conducted using plasma samples obtained from Korean patients diagnosed with *EGFR*-positive NSCLC. To complement previous study results, the present study aimed not only to evaluate the analytical performance of the Oncomine Lung cfDNA Assay compared with the cobas^®^ *EGFR* Mutation Test v2 but also the consistency of *EGFR* mutations in primary tumor tissues and plasma samples.

## 2. Materials and Methods

### 2.1. Preparation of Reference Materials and Clinical Samples

Two commercially available reference materials were used to validate the analytical performance. The SNVs and indels of the *EGFR* mutation were validated using Seraseq ctDNA *EGFR* Panel Mutation Mix Reference Materials (Item No. 0710-0703 for a variant allele frequency (VAF) of 1%; 0710-0704 for a VAF of 0.1%; SeraCare, Milford, MA, USA), which carry four common *EGFR* mutations, including p.G719S, p.E746_A750delELREA, p.T790M, and p.L858R. The SNVs and indels of the hotspot locations in driver genes targeted by the Oncomine Lung cfDNA Assay were validated using Seraseq Circulating Tumor DNA-I Reference Materials (0710-0017 for a wild-type as a negative control; 0710-0016 for a VAF of 0.1%; 0710-0014 for a VAF of 1.2%; SeraCare), which carry a single *BRAF* mutation (p.V600E), three *EGFR* mutations (p.E746_A750del, p.D770_N771insG, and p.T790M), one *KIT* mutation (p. D816V), one *KRAS* mutation (p.G12D), one *NRAS* mutation (p.Q61R), and two *PIK3CA* mutations (p.H1047R and p.N1068fs*4). The reference materials at a concentration of 20 ng/µL were used for library preparation.

A comparative evaluation of the two assays was performed using plasma samples from 16 patients diagnosed with lung cancer carrying *EGFR*-positive mutations. These mutations were identified using the PANAMutyper R *EGFR* test (PANAGENE, Daejeon, Republic of Korea) in tumor samples at initial diagnosis or in the cobas^®^ *EGFR* Mutation Test v2 in follow-up plasma samples. Tumor tissues were collected and retrieved as FFPE sides. Maxwell 16 FFPE Tissue LEV DNA Purification Kit (Promega, Madison, WI, USA) was used to extract tumor DNA for the PANAMutyper R *EGFR* test. Plasma samples were separated initially by double centrifugation (1600× *g* for 15 min, followed by 16,000× *g* for 10 min) using the Cell-Free DNA Collection tube (Roche Molecular Systems). After the completion of the routine qualitative plasma *EGFR* mutation test, the residual samples were stored in a deep freezer (−70 °C). The cfDNA was extracted using a cobas^®^ DNA Sample Preparation Kit (Roche Molecular Systems). The DNA size distribution and concentration were determined using a Qubit 2.0 Fluorometer (Thermo Fisher Scientific) and a Tapestation (Agilent, Santa Clara, CA, USA), respectively. The cfDNA libraries were constructed using a maximum input volume of 10 µL out of 40 µL eluted samples, as recommended by the manufacturer, when the total volume of cfDNA was not greater than 50 ng.

### 2.2. Real-Time Polymerase Chain Reaction for the Detection of EGFR Mutations

In the PANAMutyper R *EGFR* test, 5 μL of DNA was added to 20 μL of PCR reagent, consisting of a mixture of 19 μL of peptide nucleic acid (PNA) probe and 1 μL of Taq DNA polymerase. RT-PCR was carried out as described previously using the CFX96 Real-Time PCR Detection System (Bio-Rad, Hercules, CA, USA) [13]. The PCR-generated melting curves for each specific *EGFR* mutation were assessed based on the melting temperature (Tm) and the specific fluorescence of the melting curves. The cobas^®^ *EGFR* Mutation Test v2 was conducted by setting the DNA levels to 2 ng/μL, which were identified using a pre-defined workflow channel in a cobas^®^ z 480 analyzer (Roche Molecular Systems). Both analyses were carried out according to the manufacturers’ instructions.

### 2.3. Library Preparation, Sequencing, and Variant Calling of the Oncomine Lung cfDNA Assay

The Oncomine Lung cfDNA Assay was performed to prepare the cfDNA library by tagging each of the DNA fragments with a unique molecular barcode. Three libraries were loaded using an Ion Chef (Thermo Fisher Scientific) on an Ion 530 kit. The Ion S5 XL System (Thermo Fisher Scientific) was used to simultaneously sequence one to two Ion 530 chips. During three consecutive sequencing runs, a total of 16 libraries were sequenced and two 530 chips were analyzed at each sequencing run. Sequence alignment to the human reference genome hg19 and variant calling was performed using Torrent Suite 5.10.1 (Thermo Fisher Scientific). An Ion Reporter Software 5.10 (Thermo Fisher Scientific) was utilized for variant annotation. The default cutoff settings were applied for various parameters. Briefly, to represent the number of DNA molecules, a molecular coverage of at least 3 with a minimum identification cutoff of 0.065% must be satisfied for SNV/indel variant calling. A variant was called based on the “analysis visualization” in the Ion Reporter where the *TP53* loss of function variant with a molecular frequency < 1% was filtered out further if the variant was not defined as a hotspot mutation.

### 2.4. Statistical Analysis

The *EGFR* mutation status determined using the PANAMutyper R *EGFR* test of the paired tissue samples represented the reference method. MedCalc (version 19.5.3.; MedCalc Software, Ostend, Belgium) was used to calculate specificity, sensitivity, percentage positive agreement (PPA), percentage negative agreement (NPA), and the two-sided 95% confidence interval (CI).

## 3. Results

### 3.1. Quality Control MATRICES

The mean depth of coverage ranged from 69,000× to 105,000×. An average of 3.9 million reads was mapped to the reference genome per library, and a target relative to the designed bed file was 94.8% of the mapped reads. The uniformity of each library was 99%, which is the percentage of amplicons (bases) covered with >20% of the mean amplicons (bases).

### 3.2. Analytical Sensitivity of Plasma EGFR Mutations

The analytical sensitivity of plasma *EGFR* mutations was estimated at two different levels of VAF using the Seraseq ctDNA *EGFR* Panel Mutation Mix Reference Materials. With 20 ng of input cfDNA, the analytical sensitivities at VAFs of 1% and 0.1% were 100% and 100%, respectively. With VAFs of 1% and 0.1%, all four *EGFR* mutations, including p.G719S, p.E746_A750delELREA, p.T790M, and p.L858R, were identified by both assays in triplicate independent experiments (Table 1).

### 3.3. Analytical Sensitivity and Specificity of the Oncomine Lung cfDNA Assay

The analytical sensitivity and specificity of the Oncomine Lung cfDNA Assay was estimated with a different VAF using the Seraseq Circulating Tumor DNA-I Reference Materials. With VAFs of 1.2% and 0.1%, using 20 ng of input cfDNA, seven out of nine different mutations, including p.V600E of the *BRAF*; p.E746_A750del, p.D770_N771insG, and p.T790M of the *EGFR*; p.G12D of the *KRAS*; p.Q61R of the *NRAS*; and p.H1047R of the *PIK3CA*, were identified in the Oncomine Lung cfDNA Assay in triplicate independent experiments. Among nine mutations, p.D816V of the *KIT* and p.N1068fs*4 of the *PIK3CA* were not detected because of the non-target site. No mutation was detected in the wild-type reference material (Table 2).

### 3.4. Comparison of the Cobas^®^ EGFR Mutation Test v2 and the Oncomine Lung cfDNA Assay

When sixteen leftover plasma samples with 20 ng of input cfDNA were used, the *EGFR* mutations detected in the tumor sample at initial diagnosis were also identified in the cobas^®^ *EGFR* Mutation Test v2 and the Oncomine Lung cfDNA Assay in nine follow-up plasma samples but not in four out of sixteen patients with lung cancer. Interestingly, in three patients (D070, D453, and D076) carrying no *EGFR* mutations at initial diagnosis in the tumor sample, different *EGFR* but also *PIK3CA* and/or *TP53* mutations were detected in the cobas^®^ *EGFR* Mutation Test v2 and/or the Oncomine Lung cfDNA Assay in the follow-up plasma samples. However, all *EGFR* mutations identified in the cobas^®^ *EGFR* Mutation Test v2 were also detected in the Oncomine Lung cfDNA Assay in 12 out of 16 patients with lung cancer. Interestingly, additional minor *EGFR* mutations were only identified in the Oncomine Lung cfDNA Assay in five out of sixteen clinical samples: p.T790M (0.43%) in D096, p.C797S (0.39%) in D070, p.T790M (0.20%) in D760, p.T790M (0.25%) in D453, and p.T790M (0.43%) in D076. Furthermore, various *PIK3CA* and/or *TP53* mutations were identified together in seven out of sixteen patients with lung cancer: p.C277F of the *TP53* (8.22%) in D279, p.R248L of the *TP53* (3.37%) in D416, p.G154V of the *TP53* (71.66%) in D045, p.E542K of the *PIK3CA* (8.26%) and p.R249T of the *TP53* (43.87%) in D070, p.E545K of the *PIK3CA* (1.97%) in D453, p.R248Q of the *TP53* (1.61%) in D076, and p.R273H of the *TP53* (1.44%) in D372 (Table 3).

### 3.5. Comparison of EGFR Mutational Status of Tumor Tissues and Plasma Samples

Based on their clinical utility, we proposed that ctDNA assays for plasma *EGFR* mutation can be used for cancer screening by comparing the *EGFR* mutational status of tumor samples at the initial diagnosis and the status of plasma samples at 1-month follow-up. As a result, *EGFR* mutations were identified in thirteen (81%, 13/16) of sixteen tumor tissue samples, which included eight (61%, 8/13) samples carrying a single *EGFR* p.L858R mutation, three (23%, 3/13) samples containing a single *EGFR* exon 19 deletion, and one (8%, 1/13) sample harboring a single *EGFR* p.L861Q mutation. A single tumor tissue sample (8%, 1/13) revealed multiple mutations, including both *EGFR* exon 19 deletion and p.T790M mutations. *EGFR* mutations were identified in twelve (75%, 12/16) plasma samples in both cfDNA assays, including five (42%, 5/12) samples carrying a single *EGFR* p.L858R mutation and four (33%, 4/12) samples with single *EGFR* exon 19 deletion. Three (25%, 3/12) plasma samples carried multiple mutations, including two (67%, 2/3) samples with both *EGFR* exon 19 deletion and p.T790M mutations and one sample revealing both *EGFR* p.L858R and p.T790M mutations.

Compared with the corresponding tumor tissues, the analytical sensitivity for the detection of *EGFR* mutations in plasma samples by both cfDNA assays was 69.2% (with a 95% confidence interval (CI, 38.6–90.9%)). The PPV was 75% (95% CI, 42.8–94.5%). The analytical specificity and NPV were unknown because *EGFR*-negative samples were not evaluated. Both cfDNA assays revealed that nine of the sixteen corresponding samples carried consistent *EGFR* mutations in the plasma and the corresponding tumor tissues. The overall consistency of the *EGFR* mutations was 56.3%. Four patients (D045, D989, D270, and D372) with documented *EGFR* mutations in tumor tissue carried no detectable *EGFR* mutations in the corresponding plasma samples using both cfDNA assays. Three patients (D070, D453, and D076) with plasma *EGFR* mutations carried no identical mutations in the corresponding tumor tissue samples. One patient (D627) harbored an *EGFR* mutation in both the tumor tissue and the plasma sample. However, the findings were inconsistent in the mutation sites, which revealed a single p.L858R mutation only in the tumor tissue but both *EGFR* p.L858R and p.T790M mutations in the plasma samples. Interestingly, minor *EGFR* mutations with VAF < 1% were identified additionally in the Oncomine Lung cfDNA Assay only in five patients (D096, D070, D760, D453, and D076). The *EGFR* mutations in tumor tissues and plasma samples are presented in Table 3.

## 4. Discussion

In the present study, we performed a comparative evaluation of the Oncomine Lung cfDNA Assay and the cobas^®^ *EGFR* Mutation Test v2 to determine the precise genomic status of patients diagnosed with NSCLC. The evaluation of mutations with different VAFs under similar input volume of 20 ng was based on standard reference materials, because analytical sensitivity mainly depends on the number of VAFs. A typical 10 mL volume of blood yields on average 4 mL plasma containing 12 × 10^3^ molecules per gene, which implies a theoretical sensitivity limit of ~0.01%. If the VAF of ctDNA corresponds to 0.1%, an average of six molecules per tube carrying the respective mutation can be obtained, which may be influenced by stochastic sampling. Based on a comparison of liquid biopsies for the potential testing of cancer mutations, a ctDNA limit of detection (LoD) of <0.01% is needed for early cancer detection, whereas a ctDNA LoD of <0.1% is required for tumor liquid biopsy during disease monitoring or mutational genotyping [14].Thus, specialized and sophisticated technologies are required for the accurate, reliable, and reproducible identification of these mutations [15]. For example, the integration of NGS with unique molecular identifiers facilitates the quantitation and broad detection of rare DNA mutations with an LoD around 0.1% VAF [16]. In this study, the analytical sensitivities were 100% when the VAFs were 0.1% and 1% with 20 ng of input. In a previous study comparing identical changes in both plasma and tumor samples [17], 90 of 132 alterations were concordant (68%) for patients in early stages (stages I and II); however, 65 of 84 variants were concordant (77%) among patients at an advanced stage (stages III and IV). Further, 70 of the 75 alterations (93%) with a VAF > 1% in the plasma were identified in the tumor tissue of the same individual. Similarly, higher VAF levels were related to a significantly worse overall survival and in metastatic cancers, when determining the association of OS with maximum VAF (Max VAF) by quartiles, such as Max VAF with 0.3% for Q1, 1.3% for Q2, and 8.6% for Q3 [18]. Thus, NGS with an LoD around 1% VAF for ctDNA detection indicates the suitability of this assay for patients diagnosed with cancers at an advanced stage or metastasis.

Analytical sensitivity did not show statistically significant differences according to *EGFR* mutation type in this study, even though NGS is less sensitive for the identification of duplication, insertion, or deletions than for SNVs [19]. In Asian patients with advanced NSCLC histology, the most frequent *EGFR* mutations were exon 19 deletions (24.6%) and L858R (22.8%) missense mutations [20]. In this study, the *EGFR* p.E746_A750delELREA mutation was consistently identified with a VAF of 0.1% and 1% in triplicate independent experiments using *EGFR* panel mutation mix reference materials, even though the mean semi-quantitative index (SQI) based on the cobas^®^ *EGFR* Mutation Test v2 and the mean VAF % based on the Oncomine Lung cfDNA Assay were lower than those of other missense *EGFR* mutations. Similarly, the *EGFR* p.L858R mutation was also not inferior to other *EGFR* recurrent missense mutations, such as p.G719S and p.T790M. However, in a recent study with the same S5 platform using the Oncomine Pan-Cancer Cell-Free Assay, the reproducibility of detection of the *EGFR* p.L858R mutation was relatively low with a VAF of 1% and the identification of the *EGFR* p.L858R mutation with a VAF of 0.5% was less sensitive, despite adequate coverage. Thus, the increased recurrence of *EGFR* mutations in the Asian population suggests the need for enhanced awareness of issues related to the precision and/or sensitivity of *EGFR* mutation testing, because the analytical performance of the *EGFR* mutation test can differ even with the same assay or the same platform [12].

Comparative results reported by independent studies may be useful in estimating the analytical performance of the assay, which can differ even with similar sample types, reagents, and/or the platform. In the comparative evaluation using the reference material, the cobas^®^ *EGFR* Mutation Test v2 and the Oncomine Lung cfDNA Assay showed concordance. Both assays revealed 100% of the *EGFR* mutations with VAFs of 1% and 0.1% using 20 ng of input DNA and showed a decrease in the mean SQI and VAF % with the same input. In previous Korean studies, the NGS-based platforms (Oncomine Lung cfDNA and Pan-Cancer Cell-Free Assays) were more sensitive than the cobas^®^ *EGFR* Mutation Test v2 for the detection of mutations with a VAF of 0.1 [11,12]. However, the discrepancy in the detection rate between the current and previous studies might be attributed to variable amounts of input nucleic acid. The previous study using the Oncomine Lung cfDNA Assay as the reagent with the same platform showed a sensitivity of 81% with <20 ng of cfDNA at a VAF of 0.1% [21]. However, So and her colleagues [12] reported a sensitivity of 50% under the same conditions using the Oncomine Pan-Cancer Cell-Free Assay with a different reagent using the same platform. The same assay showed a sensitivity of 80% for SNV with a VAF of 0.1% in the study undertaken by the manufacturer [22].

The liquid biopsy of plasma samples showed higher specificity for the detection of *EGFR* mutations in patients with NSCLC, whereas tissue biopsy was still required for the evaluation of patients with negative liquid biopsy due to its lower sensitivity [23]. Plasma cfDNA testing is a feasible and sensitive option for the identification of *EGFR* mutations and represents an alternative surrogate marker [24]. Two meta-analyses demonstrated a pooled specificity of 93.5–98% and a sensitivity of 67.4–68% for cfDNA, when plasma samples were tested to determine the *EGFR* mutational status compared with matched tumor tissues [23,25]. The cobas^®^ *EGFR* Mutation Test v2 is easy to operate and stable for the screening of plasma *EGFR* mutations in routine clinical settings [3,26], compared with the limitations associated with NGS in liquid biopsy, such as limited equipment availability, cumbersome assay, and a long turnaround time [27]. However, RT-PCR or emulsion PCR techniques, such as digital Bead Emulsion Amplification and Magnetic (BEAMing) and ddPCR, are not suitable for analyzing the complex and multiple genomic aberrations emerging as relevant targets for precision medicine in NSCLC [28]. In our study, a moderate sensitivity of 69.2% was achieved for identifying plasma *EGFR* mutations via both cfDNA assays, consistent with previous studies [23,25], which suggests that plasma *EGFR* mutations might be highly predictive of identical mutations in corresponding tumor tissues. Furthermore, various *PIK3CA* and/or *TP53* mutations were identified only via Oncomine Lung cfDNA Assay in this study. NGS panels, such as the Oncomine Lung cfDNA Assay, facilitated the detection of novel and multiple resistance aberrations, suggesting the substantial heterogeneity of variant types and gene mutations before and after the acquisition of drug resistance. The cfDNA NGS assay can be utilized specifically to determine the effectiveness of molecular target therapy when multiple different mutations can be traced in the peripheral blood of patients.

However, *EGFR* mutations were detected inconsistently, with an overall consistency of 56.3% between the tumor tissue and the corresponding plasma samples in seven patients. The PANAMutyper R *EGFR* test showed an analytical sensitivity of 1% during the analysis of the corresponding tumor tissue samples. In addition to the analytical sensitivity of the assay, the prolonged storage of blood samples may affect the detection efficiency of the plasma *EGFR* mutations [29]. In addition, the low input of cfDNA template in the reaction may be affected by the low level of cfDNA extracted from plasma samples, thus leading to *EGFR* mutation detection [30]. Further, intratumor heterogeneity and the relatively low sensitivity of the PANAMutyper R *EGFR* test contribute to the discordant *EGFR* mutation status. Because of the molecular heterogeneity of tumors, the complete genomic landscape may not be captured when sampling a single lesion [31]. In particular, minor *EGFR* mutations with a VAF <1% were identified additionally in the Oncomine Lung cfDNA Assay involving five patients, even though multiple *EGFR* mutations are a rare event with a frequency of <1% (446 of 46,679 patients), which suggests that a majority of NSCLC patients carry single *EGFR* mutations in clinical encounters [32]. Acquired and de novo *EGFR* p.T790M mutations were the most recurrent single mutations. Additionally, *EGFR* p.L858R with p.T790M and p.T790M with exon 19 deletions were the most common double mutations [32]. Since detection sensitivity using NGS reached the 0.1% level, the frequency of detection of multiple *EGFR* mutations will increase, suggesting the need for further investigation into its clinical utility.

The present study has some limitations. First, most of the analysis involved clinical samples and reference materials containing mainly *EGFR* mutations, which included other driver genes. Other clinically significant mutations, such as CNV or structural variants, were not estimated as they were not considered for mutational targeting in the Oncomine Lung cfDNA Assay. Further sophisticated studies are required to determine the analytical validity of the Oncomine Lung cfDNA Assay, because the test performance based on clinical samples may not be similar to that of the reference materials. Second, a more comprehensive Oncomine Assay has been introduced and its clinical validity has been reported in various solid cancers, lung cancer [12], colorectal cancer [33], cancers of the central nervous system [34], and endometrial cancer [35]. The clinical validity of the assay in various cancer types remains to be determined. Thus, the use of the Oncomine Lung cfDNA Assay for lung cancer alone has practical limitations. Third, the sample size in this study was small, even though different reference materials and clinical samples were used. Further studies with large sample sizes are required.

## 5. Conclusions

The Oncomine Lung cfDNA Assay can be used to identify plasma *EGFR* mutations in patients with NSCLC, although further large-scale studies are needed to evaluate the analytical validity for other types of aberrations and genes using clinical samples. Medical laboratories should evaluate the characteristics and performance of analytical assays before utilizing them in the analysis of clinical specimens, because analytical sensitivity varies with sample quality, methods used to handle samples, and variant types.

## Figures and Tables

**Table 1:** Comparison of analytical sensitivity between the Cobas *EGFR* Mutation Test v2 and the Oncomine Lung cfDNA Assay in Seraseq ctDNA *EGFR* Panel Mutation Mix Reference Materials.

*EGFR* Mutation	AF 1%		AF 0.1%	
	Cobas (Mean SQI)	Oncomine (Mean VAF %)	Cobas (Mean SQI)	Oncomine (Mean VAF %)
p.G719S	Detected (11.43)	Detected (1.21)	Detected (5.97)	Detected (0.16)
p.E746_A750delELREA	Detected (9.86)	Detected (1.01)	Detected (4.22)	Detected (0.11)
p.T790M	Detected (10.31)	Detected (1.14)	Detected (5.86)	Detected (0.15)
p.L858R	Detected (10.96)	Detected (1.16)	Detected (5.49)	Detected (0.14)

AF, allele frequency; SQI, semiquantitative index; VAF, variant allele frequency.

**Table 2:** Analytical sensitivity and specificity of the Oncomine Lung cfDNA Assay in Seraseq Circulating Tumor DNA-I Reference Materials.

Gene	Mutation	AF 1.2% (Mean VAF %)	AF 0.1% (Mean VAF %)	Wild-Type
*BRAF*	p.V600E	Detected (1.57)	Detected (0.13)	Not Detected
*EGFR*	p.E746_A750del	Detected (1.22)	Detected (0.10)	Not Detected
*EGFR*	p.D770_N771insG	Detected (1.32)	Detected (0.11)	Not Detected
*EGFR*	p.T790M	Detected (1.75)	Detected (0.15)	Not Detected
*KIT* *	p.D816V	Not available	Not available	Not available
*KRAS*	p.G12D	Detected (2.09)	Detected (0.14)	Not Detected
*NRAS*	p.Q61R	Detected (1.92)	Detected (0.13)	Not Detected
*PIK3CA*	p.H1047R	Detected (1.69)	Detected (0.17)	Not Detected
*PIK3CA* *	p.N1068fs*4	Not available	Not available	Not available

* Non-target site. AF, allele frequency; VAF, variant allele frequency.

**Table 3:** Comparison between the cobas^®^ *EGFR* Mutation Test v2 and the Oncomine Lung cfDNA Assay in 16 patients diagnosed with lung cancer.

Patients	Tumor Sample	Plasma Sample			
	PANAMutyper	Cobas (SQI)	Oncomine (VAF %)		
	*EGFR*	*EGFR*	*EGFR*	*PIK3CA*	*TP53*
D279	p.L858R	p.L858R (11.03)	p.L858R (7.34)		p.C277F (8.22)
D788	p.E746_A750del	Ex19Del (10.04)	p.E746_A750del (1.48)		
D416	p.E746_A750del, p.T790M	Ex19Del (13.79), p.T790M (8.72)	p.E746_A750del (3.56), p.T790M (2.63)		p.R248L (3.37)
D627	p.L858R	p.L858R (10.46), p.T790M (10.39)	p.L858R (5.22), p.T790M (2.29)		
D619	p.L858R	p.L858R (9.21)	p.L858R (3.18)		
D783	p.L858R	p.L858R (5.01)	p.L858R (1.01)		
D045	p.L858R	Not Detected	Not Detected		p.G154V (71.66)
D096	p.L747_A750delinsP	Ex19Del (13.35)	p.L747_A750delinsP (3.82), p.T790M (0.43)		
D989	p.L858R	Not Detected	Not Detected		
D070	Not Detected	Ex19Del (21.38) p.T790M (16.31)	p.E746_A750del (54.65), p.T790M (19.67) p.C797S (0.39)	p.E542K (8.26)	p.R249T (43.87)
D871	p.E746_A750del	Ex19Del (14.97)	p.E746_A750del (8.63)		
D270	p.L861Q	Not Detected	Not Detected		
D760	p.L858R	p.L858R (8.27)	p.L858R (2.37), p.T790M (0.20)		
D453	Not Detected	Ex19Del (12.06)	p.E746_T751delinsA (1.50), p.T790M (0.25)	p.E545K (1.97)	
D076	Not Detected	p.L858R (15.19)	p.L858R (17.73), p.T790M (0.43)		p.R248Q (1.61)
D372	p.L858R	Not Detected	Not Detected		p.R273H (1.44)

SQI, semiquantitative Index; VAF, variant allele frequency.

## Data Availability

Not applicable.
